# Going too far: why universal recommendations for alcohol abstinence may not be beneficial for public health

**DOI:** 10.1093/eurpub/ckae192

**Published:** 2025-04-03

**Authors:** Kevin Shield, Saverio Stranges

**Affiliations:** Centre for Addiction and Mental Health, Institute for Mental Health Policy Research, Toronto, ON, Canada; Dalla Lana School of Public Health, University of Toronto, Toronto, ON, Canada; Department of Epidemiology & Biostatistics, Schulich School of Medicine & Dentistry, Western University, London, ON, Canada; Department of Epidemiology & Biostatistics, Schulich School of Medicine & Dentistry, Western University, London, ON, Canada; Department of Family Medicine, Schulich School of Medicine & Dentistry, Western University, London, ON, Canada; Department of Medicine, Schulich School of Medicine & Dentistry, Western University, London, ON, Canada; Department of Clinical Medicine and Surgery, University of Naples Federico II, Naples, Italy

Guidance on alcohol and health varies substantially depending on the government or organization issuing the recommendations [1]. The Nordic Nutrition Recommendations 2023, and the governments of Latvia and Lithuania advise that everyone should avoid alcohol consumption [1]. This raises two questions: How do organizations arrive at the conclusion that no one should consume alcohol? And, is it in public health’s best interest to recommend total abstinence?

While there is consensus drinking to a blood alcohol content of ≥0.03 g/dL is risky as it leads to an increase in the risk of injuries [2]. Evidence on the health impact of consuming alcohol over a longer period of time (i.e. weeks) is less clear. In explaining their stance, the Nordic Council of Ministers referenced the World Health Organization’s (WHO) claim that no level of alcohol consumption is safe [3]. This assertion stems from the fact there is no level of alcohol intake that offers both protection against cardiovascular diseases or type 2 diabetes and does not increase cancer risk [3, 4]. This position aligns with the precautionary principle, which asserts that the lack of evidence proving that low levels of alcohol consumption are risk-free justifies recommending abstinence. This approach contrasts with that of most health guidelines, which offer risk-based information to the general population based on the point estimate of alcohol’s impact on health.

Because there is insufficient evidence of an overall protective health effect of alcohol, most public health agencies agree that no one should start drinking alcohol for health reasons. However, should people who already consume one drink per week be advised to abstain? Current guidelines do not suggest that these individuals reduce their alcohol consumption. This is partly due to the social acceptance of the voluntary risks. Furthermore, based on ‘2023 Canadian Guidance on Alcohol and Health’, there is a non-significant protective effect of consuming one drink per week on health and, therefore, the impact of alcohol and health at this level is unknown [2]. Current guidelines on alcohol and health account for existing consumption patterns, including the tendency of some regular drinkers to engage in heavy episodic drinking (HED), when formulating recommendations for weekly consumption. However, if these guidelines were based on the assumption that current regular drinkers do not engage in HED, the protective effects of alcohol on cardiovascular diseases would likely be more pronounced.

The precautionary principle also influences how alcohol-related risks are communicated. For example, the causal links between alcohol and diseases like pancreatic and stomach cancers remain unclear, and these potential risks would be emphasized within a precautionary framework. Additionally, the protective effect of small amounts of alcohol in reducing the risk of cardiovascular diseases—has been questioned, and this uncertainty would be communicated in a precautionary regime. While the precautionary principle aims to prioritize public health by advocating for abstinence in the face of uncertainty about alcohol’s effects, this messaging may be perceived as alarmist, thereby potentially diminishing public receptiveness.

Large shifts in alcohol and health guidelines, which would occur when precautionary principles are applied, are often met with skepticism. For instance, the 2023 Canadian Guidance on Alcohol and Health substantially lowered the threshold for low-risk alcohol consumption, recommending no more than two drinks per week, compared to the previous guidelines of 10 drinks per week for women and 15 for men. This change in Canadian guidelines, in contrast to other countries like Australia and the UK, stems from the use of a standard low-risk definition of 1 in 1000 lifetime deaths due to alcohol, as opposed to the non-standard definition of 1 in 100 lifetime deaths [2]. Despite an editorial in the Canadian Medical Association Journal supporting the appropriateness of the 1 in 1000 lifetime death threshold, Health Canada has not adopted these guidelines, though some provinces have [5]. Similarly, the 2023 Nordic Nutrition Recommendations for abstention from alcohol have not been adopted by Nordic countries. Such substantial shifts toward abstinence or stricter low-risk consumption may seem unrealistic for a large portion of the population, depending on current drinking habits, due to a widespread social acceptance of moderate alcohol intake across several geographic contexts. For example, in Canada in 2019, 22.1% of adults abstained from alcohol, while 27.5% of drinkers consumed no more than two drinks per week [2].

Consuming alcohol—even at low levels—poses a risk, as it increases the likelihood of developing cancer. Furthermore, research shows that even low consumption can exceed the low-risk thresholds [3]. However, while this information may lead to stricter guidance on alcohol and health, it could also reduce social acceptance of what is considered “low-risk” alcohol consumption. Similar to the shift in attitudes toward tobacco smoking, changing the social narrative around the harms of low-volume alcohol consumption may take time. That said, current scientific evidence is insufficient to conclude that consuming one drink per week has a net detrimental impact on health, hence public health recommendations for complete abstinence seem not to be fully justified at the present time.

Conflict of interest: None declared.
